# The 140 years' journey of gastric cancer surgery: From the two hands of Billroth to the multiple hands of the robot

**DOI:** 10.1002/ags3.12442

**Published:** 2021-02-12

**Authors:** Masanori Terashima

**Affiliations:** ^1^ Division of Gastric Surgery Shizuoka Cancer Center Nagaizumi Japan

**Keywords:** extended surgery, gastric cancer, laparoscopic gastrectomy, postoperative complications, robotic gastrectomy

## Abstract

After the initial achievement by Billroth in 1881, surgery for gastric cancer has become increasingly extended. However, it turned out to be limited in Western countries after the publication that denied the role of extended surgery in the 1960s. While surgeons in Japan were still enthusiastic about extended surgery, the Japan Clinical Oncology Group (JCOG) conducted clinical trials to validate the role of extended surgery. Contrary to expectations, the efficacy of extended surgery was not demonstrated. In gastric cancer surgery, postoperative complications were reported to be associated with poor survival. A survival benefit could not be obtained by extended surgery, with high morbidity. Therefore, the paradigm had been changed from extended surgery to minimally invasive surgery (MIS). As an MIS for gastric cancer, laparoscopic surgery has been considered a practical method. Initial laparoscopic gastrectomy (LG) was first performed by Kitano in 1991. Thereafter, LG became increasingly common. Several clinical trials demonstrated the noninferiority of LG to open gastrectomy. LG is now regarded as the standard for cStage I gastric cancer, and the indication is expanding to advanced cancer. However, LG has some drawbacks owing to the restriction of movement caused by straight‐shaped forceps. Robotic gastrectomy (RG) is considered a major breakthrough to circumvent the drawbacks in LG using articulated devices. However, the solid evidence demonstrating the advantage of RG has not been proved yet. The JCOG is now conducting a randomized controlled trial to evaluate the superiority of RG to LG in terms of reducing morbidity.

## 1 INTRODUCTION

The basis of surgery for gastric cancer is gastrectomy and lymph node dissection. Initially, the effectiveness of extended surgery was shown to improve the therapeutic results; however, the attempts failed to demonstrate its usefulness. After many twists and turns, D2 dissection is now regarded as the standard treatment for advanced gastric cancer.

On the other hand, the effectiveness of minimally invasive surgery (MIS) has been verified, and MIS is now positioned as the standard treatment for early gastric cancer. Furthermore, in recent years the effectiveness of robotic surgery has been confirmed.

Likewise, gastric cancer surgery has changed on the basis of the results of clinical trials, and establishing a standard treatment based on scientific evidence is extremely important for the future. The history of gastric cancer surgery is reviewed in this article.

## 2 THE DAWN OF GASTRIC CANCER SURGERY

The major history of gastric cancer is shown in Table 1. Since the initial achievement by Billroth in 1881,^1^ many surgeons have tried to perform gastrectomy for gastric cancer. However, at that time, as perioperative management such as general anesthesia and infectious disease control had not been established, the mortality was extremely high. The mortality rate reported by Billroth himself from 1878 to 1890 was 55.2% (16/29 cases),^2^ and that reported by Billroth's progeny Mikulicz from Breslau University from 1882 to 1895 was 27.8% (5/18 cases).^3^

**TABLE 1:** Major history of gastric cancer surgery

	West		Japan
1881	Billroth succeeded in gastrectomy		
1897	Schlatter succeeded in total gastrectomy		
		1897	Kondo succeeded in gastrectomy
1903	Mikulicz–Radecki performed pancreatectomy		
		1905	Kitagawa succeeded in total gastrectomy
1910	Grove proposed bursectomy		
		1928	Miyake published “gastric cancer”
		1942	Kajitani proposed extended dissection
1948	Brunschwig performed pancreatosplenectomy		
1950	Lahey proposed total gastrectomy for EGC		
1953	Appleby proposed the Appleby operation		
1960	Lawrence denied extended surgery		
		1962	JGCA was founded
	Trends for limited surgery		The Japanese classification was published
1990	The MRC and Dutch trials were started		
		1991	Kitano performed laparoscopic gastrectomy
		1995	JCOG9501 and JCOG9502 were started
		2001	JCOG0110 was started
		2002	Hashizume performed robotic gastrectomy
		2009	JCOG0912 was started
2010	Results of the 15‐year follow‐up of the Dutch trial were published		
		2019	JCOG1907 was started

Thereafter, Mikulicz showed four patterns of progression in gastric cancer, namely, local progression, lymphatic progression, hematogenous progression, and peritoneal dissemination. Lymph node dissection is extremely important to cure gastric cancer.^4^ In addition, the extent of resection had been expanded to improve the curability of gastric cancer. Mikulicz reported the results of 30 patients who underwent combined resection of the pancreas for pancreatic invasion from gastric cancer in 1903.^5^ Mikulicz had already mentioned pancreatic fistula after pancreatic injury. The mortality rate was 27.5% (25/91 patients) without pancreatic resection and 70% (21/30 patients) with pancreatic resection. The cause of death was peritonitis in all 21 patients. Meanwhile, as the safety of surgery had increased owing to the advances in anesthesia and other perioperative management, surgery for gastric cancer had been steered toward extended surgery.

## 3 Direction Toward Extended Surgery

In 1910, Groves, of the Bristol Hospital in the UK, proposed that resection of the omentum (partially the omental bursa) was necessary for a secure lymph node dissection,^6^ after which omentectomy and bursectomy became widespread worldwide. Brunschwig,^7^ of the Memorial Hospital for Cancer and Allied Diseases, New York, reported the results of combined resection of the pancreas and spleen for the purpose of lymph node dissection for gastric cancer. Total gastrectomy with pancreato‐splenectomy was performed in 14 patients. Only two patients died from surgery, and the safety was confirmed.^7^ Lahey, of the Lahey Clinic, Boston, recommended total gastrectomy even for the lower part of gastric cancer because of the concept that gastric cancer progresses through lymphatic vessels in the stomach wall.^8^ The surgical mortality rate was reduced from 34.6% to 9.4% in the latter stage, and the 5‐year survival rate was as high as 12.5%, which was a sufficient result at that time.

Appleby^9^ surgery is the ultimate extended surgery. Mikulicz‐Radecki had already emphasized the importance of suprapancreatic lymph node dissection in gastric cancer at the beginning of the 20th century. To secure the suprapancreatic lymph node dissection, Appleby, from Vancouver, Canada, proposed the so‐called Appleby operation, in which the celiac artery is ligated at the root.^9^ The operation was performed in 13 patients, and operative mortality occurred in only one patient, which suggests the safety of the procedure. Thereby, extended surgery has been actively performed for gastric cancer, mainly in the US.

## 4 Turning Point

Lawrence and McNeer^10^ of the Memorial Center for Cancer and Allied Diseases, New York, compared the results of extended surgery since 1951 (total gastrectomy, pancreatosplenectomy, and extensive omental resection) with those of previously performed distal gastrectomy. They reported no significant difference in survival.^10^ Later, from the theory that lymph node metastasis is an indicator of systemic disease^11^ and the results of a randomized controlled trial that proved that lymph node dissection did not improve survival,^12^ systematic lymph node dissection has not been used in breast cancer. Gastric cancer is thought to have similar biological characteristics, and systematic lymph node dissection has gradually ceased in Europe and the US.

In Japan, lymph node metastasis was considered a local disease, and surgical removal of the regional lymph node was believed to lead to improvement of treatment results. In 1962, the Japanese Research Society for Gastric Cancer was established, and the Japanese Classification for Gastric Cancer was published. Lymph nodes were numbered,^13^ and the national registration was also started. As data have shown the importance of lymph node dissection and the treatment results have demonstrated the effectiveness of extended dissection, surgery has become increasingly expanded. Even para‐aortic lymph nodes are considered the target of dissection, and some specialized institutions have reported better survival due to para‐aortic lymph node dissection.^14^ In these periods, a huge discrepancy existed between Japan and the Western countries.

## 5 CLINICAL TRIALS FOR GASTRIC CANCER SURGERY AND ESTABLISHMENT OF A STANDARD TREATMENT

The major clinical trials conducted for gastric cancer are shown in Table 2. In the 1980s, two randomized controlled trials were conducted in Europe to verify the effectiveness of Japanese‐style D2 lymph node dissection. One was the Medical Research Council ST01 trial conducted in the UK,^15^ and the other was the Dutch trial conducted in the Netherlands.^16^ Although large differences in quality assurance exist between the trials, the results were generally comparable, with a high rate of postoperative complications and surgical deaths in the D2 group, and no additional survival effect of D2. Therefore, in the latter half of the 1990s, D1 dissection was positioned as the standard treatment in Europe and the US.

**TABLE 2:** Major clinical trials for gastric cancer

Trial	Year	Country	No. of patients	Subject	Intervention	Design	Endpoint	Result	Reference
MRC ST01	1999	UK	400	Stage I–III	D1 vs D2	Superiority	OS	Negative	15
Dutch D1/D2	1999	Netherlands	711	Adenocarcinoma, M0	D1 vs D2	Superiority	OS	Negative	16
JCOG9502	2006	Japan	503	Esophageal invasion ≥3 cm, M0	Abdominal vs left thoraco‐abdominal	Superiority	OS	Negative	20
JCOG9501	2008	Japan	523	T2b–4, M0, CY0	D2 vs D2 + PAND	Superiority	OS	Negative	17
JCOG0110	2017	Japan	503	T2‐4, M0, CY0, w/o GC invasion	Splenectomy vs spleen preservation	Noninferiority	OS	Positive	19
JCO1001	2018	Japan	1204	T3‐4, M0	Omentectomy vs bursectomy	Superiority	OS	Negative	21
KLASS01	2019	Korea	1416	cStage I	ODG vs LDG	Noninferiority	OS	Positive	27
JCO0912	2020	Japan	921	cStage I	ODG vs LDG	Noninferiority	RFS	Positive	26
CLASS01	2019	China	1056	cStage II–Iva	ODG vs LDG	Noninferiority	DFS	Positive	30
KALSS02	2020	Korea	1050	cStage II–Iva	ODG vs LDG	Noninferiority	RFS	Positive	31
STOMACH	2020	Netherlands	96	T1‐3, N0‐1, Mo	OTG vs LTG after NAC	Noninferiority	Extent of LN dissection	Positive	34

DFS, disease‐free survival; LDG, laparoscopic distal gastrectomy; LN, lymph node; LTG, laparoscopic total gastrectomy; NAC, neoadjuvant chemotherapy; ODG, open distal gastrectomy; OS, overall survival; OTG, open total gastrectomy; PAND, para‐aortic lymph node dissection; RFS, relapse‐free survival.

On the contrary, local control with lymph node dissection was believed to increase the curability of cancer in Japan, so clinical trials were planned to establish the evidence of extended surgery. The JCOG9501 study, planned by the Japan Clinical Oncology Group (JCOG), was designed to validate the superiority of prophylactic para‐aortic lymph node dissection to conventional D2 dissection in advanced gastric cancer. As a result, the superiority of para‐aortic lymph node dissection was not proved, and the significance of prophylactic para‐aortic lymph node dissection was denied.^17^ This result parallels that of a recently published ovarian cancer trial investigating the role of prophylactic para‐aortic lymph node dissection.^18^ The survival benefit of prophylactic pelvic and para‐aortic lymph node dissection has not been proven yet. It has been speculated that the high incidence of morbidity and reoperation rate may have contributed to the negative results obtained in that study.

Since then, D2 dissection has been regarded as the standard treatment for advanced gastric cancer in Japan. At this period, D1 dissection was the standard treatment in the West, and D2 dissection was the standard in Japan and other Asian countries. This situation, in which the standard surgery was different between the East and the West, continued for a while.

However, the publication of the 15‐year follow‐up results of the Dutch trial in 2010 proved the significance of D2 dissection in terms of long‐term results.^19^ In addition, the safety of D2 dissection in several specialized centers in Europe has also been demonstrated. Thus, combined with the efficacy and safety report, D2 dissection has been regarded as a standard for advanced gastric cancer in Europe and the US.

At this point, the difference between the West and the East was only splenectomy. In Western countries, splenectomy was not performed unless direct infiltration into the spleen or pancreas was observed, because splenectomy increases postoperative complications and mortality. On the other hand, splenectomy has been considered essential for complete dissection of the splenic hilum lymph nodes in Japan. The JCOG0110 was conducted to confirm the noninferiority of spleen preservation to splenectomy in upper gastric cancers without greater curvature invasion.^20^ The survival was similar in both groups, and the noninferiority of spleen preservation was confirmed. On the basis of the results of this trial, spleen preservation has been regarded as the standard surgery in Japan. After the release of the results of the trial, D2 dissection without splenectomy has been considered the world standard. However, whether splenectomy is needed for proximal tumors with greater curvature invasion remains an unanswered question. Several retrospective studies have suggested the relevance of splenectomy in proximal tumors with greater curvature invasion.^21^, ^22^ Additionally, the effectiveness of splenic hilar lymph node dissection with spleen preservation has been reported.^23^ This procedure appears to be attractive not only from a safety perspective but also because of the oncological benefits. JCOG is now conducting a phase II trial evaluating the safety of laparoscopic splenic hilar lymph node dissection (UMIN000037580), and it is expected that this issue will be resolved in the near future.

## 6 Clinical Trials Conducted by the JCOG

In addition to the above‐mentioned trials, the JCOG conducted several large‐scale surgical clinical trials for advanced gastric cancer. JCOG9502 was conducted to validate the superiority of the left thoracoabdominal approach to the abdominal‐transhiatal approach for gastric cancer with esophageal invasion ≤3 cm.^24^ In the 1990s, curability was believed to improve by intensive dissection of the lower mediastinal lymph node via the left thoracoabdominal approach for gastric cancer with esophageal invasion. However, the study was terminated because of the futility at the second interim analysis, and the result was revealed. Unexpectedly, the left thoracoabdominal approach demonstrated a relatively poorer survival than the abdominal‐transhiatal approach. On the basis of the results, the abdominal‐transhiatal approach has become the standard treatment for gastric cancer with esophageal invasion ≤3 cm.

Another pivotal clinical trial was JCOG1001.^25^ For advanced gastric cancer with positive serosal invasion, gastrectomy with omento‐bursectomy had long been regarded as the standard in Japan. However, since the middle 1990s, omentectomy alone without bursectomy has become increasingly common on the basis of the small clinical and translational studies. Meanwhile, the results of the clinical trial suggesting the usefulness of omento‐bursectomy were published by a clinical trial group led by Osaka University. Therefore, the JCOG conducted a phase III trial to verify the superiority of omento‐bursectomy to omentectomy, which was regarded as the standard at that time. This study was also terminated at the second interim analysis because of futility and the results were revealed. The superiority of omento‐bursectomy was not demonstrated.

**FIGURE 1:**
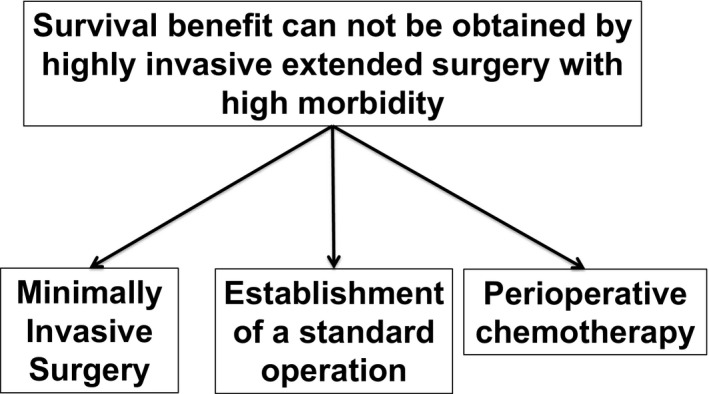
Paradigm shift for gastric cancer surgery

Considering these two trials, as well as the aforementioned JCOG9501 and JCOG0110 trials, the superiority of extended surgery has not been confirmed. Although mortality was extremely low in either trial, an increase in the postoperative complication rate was observed in the extended surgery group in all the studies. Postoperative complications have been reported to be an independent prognostic factor in gastric cancer.^26^, ^27^ We speculated that a survival benefit cannot be obtained by highly invasive surgery with high morbidity. By a curious coincidence, the contradiction of extended surgery reported by the US in the 1960s was proved more scientifically after 50 years. Since then, the paradigm had shifted from extended surgery to MIS, the establishment of a standard surgery, and the development of perioperative chemotherapy (Figure 1).

## 7 Laparoscopic Surgery

Laparoscopic gastrectomy (LG) has been developed as a practical method for MIS for gastric cancer. The world's first LG was reported by Kitano in 1991.^28^ Since then, LG has become common worldwide with the advancement in surgical instruments such as the video system, forceps, and energy devices. However, owing to the technically demanding procedure using long linear forceps, confirming the safety and oncological tolerability took time. At first, the evidence was established through clinical trials only for early gastric cancer, for which the degree of lymph node dissection is limited and handling the stomach is easy.

The JCOG conducted a phase II clinical trial in patients with cStage I gastric cancer who underwent distal gastrectomy (DG) to investigate the safety of LG in JCOG0703.^29^ The primary endpoints were the incidence of anastomotic leakage and pancreatic fistula. A total of 176 patients were enrolled, and the incidence of anastomotic leakage and pancreatic fistula were both extremely low, at 1.7%. The null hypothesis was rejected. On the basis of the results, the JCOG conducted a phase III trial to verify the noninferiority of laparoscopic distal gastrectomy (LDG) to open distal gastrectomy (ODG) for cStage I gastric cancer in JCOG0912.^30^ The noninferiority of LDG was confirmed in terms of relapse‐free survival, and laparoscopic surgery became one of the standard treatments in DG for cStage I gastric cancer. A similar noninferiority trial was conducted in South Korea (KLASS01), which also demonstrates the noninferiority of LDG to ODG.^31^ In addition, the JCOG conducted a single‐arm confirmatory phase III trial to verify the safety of laparoscopic total gastrectomy (LTG) and laparoscopic proximal gastrectomy (LPG) to expand the indications for LG in JCOG1401.^32^ As the survival of patients with cStage I gastric cancer was good and the extent of lymph node dissection may not significantly differ between DG and TG for cStage I gastric cancer, which would have a significant impact on prognosis, the results of JCOG0912 could be extrapolated for survival results once the safety of TG and PG was confirmed. Thus, a single‐arm confirmatory phase III trial was planned. A total of 245 patients were enrolled, and the incidence of anastomotic leakage at the esophago‐jejunostomy site, which was the primary endpoint, was extremely low, at 2.4%. It was significantly lower than the prespecified threshold of 8%, and the null hypothesis was rejected. The safety of LTG and LPG was verified. Similarly, a phase II study evaluating the safety of LTG was conducted in Korea (KLASS‐03), demonstrating the safety of LTG.^33^ From these results, LTG and LPG for cStage I have also become standard treatments.

On the other hand, LG for advanced gastric cancer has been considered to require further examination from the viewpoints of tumor handling, accuracy of D2 lymph node dissection, and safety. Phase III clinical trials were conducted in Japan, South Korea, and China to examine the noninferiority of LDG to ODG for advanced gastric cancer.^34^, ^35^, ^36^ The result of the primary analysis was first reported in the CLASS01 trial performed in China, which proved the noninferiority of LDG to ODG. Noninferiority was confirmed using the point estimation of 3‐year relapse‐free survival.^34^ Next, the results of the KLASS02 study in Korea were reported, and the noninferiority of LDG was also proved.^35^ The results of the JLSSG study in Japan will be reported in 2021,^36^ and these results may allow laparoscopic surgery for advanced gastric cancer as the standard treatment.

A phase III clinical trial investigating the usefulness of LG was also planned in Europe. The LOGICA trial was designed to verify the superiority of LG over open gastrectomy in terms of hospital stay,^37^ and the STOMACH trial is a noninferiority study comparing the extent of lymph node dissection in LTG over open total gastrectomy after neoadjuvant chemotherapy. The results of the STOMACH trial have already been published, and no significant difference was observed between the two groups regarding the number of lymph node dissections, which is a primary endpoint.^38^ From the results of these studies, LG is expected to be regarded as the standard treatment even in Europe and the US in the future.

Although laparoscopic surgery has become increasingly common worldwide, it is still technically demanding and requires acquisition of a certain level of skill to perform it. In Japan, a cohort study using data from the National Clinical Database, which is a nationwide registry system, is being conducted to evaluate the safety of LG in comparison with that of open gastrectomy in clinical practice. A total of four prospective and retrospective trials were conducted for DG and TG, respectively.^39^, ^40^, ^41^, ^42^ Surprisingly, laparoscopic surgery has been reported to have a higher incidence of postoperative complications than open surgery. A retrospective comparison of TG reported that the incidence of anastomotic leakage was significantly higher in LTG than in OTG.^41^ The incidence of pancreatic fistula has also been reported to be significantly higher in LDG than in ODG in both retrospective and prospective studies.^39^, ^40^ This is probably owing partly to the compression of the pancreas during suprapancreatic lymph node dissection using straight‐shaped forceps. Circumvention of the restriction of movement is a major issue in laparoscopic surgery. Another important issue with LG is the learning curve. Even among the technically certified physicians of the Japanese Society of Endoscopic Surgery, which is the only credited system for laparoscopic surgery in the world, the pass rate is in the 20% range, and the acquisition of LG techniques is considerably difficult, which cannot be denied. These points are the problems that must be solved by laparoscopic surgery.

## 8 Robotic Gastrectomy

In recent years, robot‐assisted or robotic gastrectomy (RG) has been highlighted as an MIS for gastric cancer. RG overcomes the above‐mentioned major drawbacks of LG by using forceps with an articulation of 7 degrees of freedom, a high‐resolution three‐dimensional camera, and a motion scale, and by preventing hand movement. With regard to gastric cancer, the first robot‐assisted case (robot‐assisted DG) was reported in 2002 by Hashizume et al^43^ and updated by Kakeji et al in 2006.^44^ Since then, with the spread of the da Vinci surgical system (DVSS), RG has become increasingly more common worldwide.

Several reports of meta‐analyses have compared RG and LG.^45^, ^46^, ^47^ RG has been reported to show a prolonged operation time and a slightly smaller amount of blood loss; however, no reports have suggested the absolute advantage of RG. In the report from Japan, the safety of RG was evaluated in single‐arm clinical phase II studies,^48^, ^49^ and one report indicated that RG had fewer complications by comparing RG and LG.^50^ No such report has been made by countries other than Japan. A recent meta‐analysis from Italy has reported that robotic surgery excels in short‐term results, but all the underlying articles are from Japan.^51^ In a multicenter prospective cohort study conducted in Japan, the postoperative complication rates were reported to be reduced to less than half in RG as compared with laparoscopic surgery as the historical control.^52^

While in Korea a multicenter prospective nonrandomized control study was conducted with 434 patients who underwent RG or LG.^53^ No significant difference was observed between RG (n = 223) and LG (n = 211) in the incidence of postoperative complications (11.9% vs 10.3%), and the mortality rate was 0% in both groups. However, the operative time was approximately 40 minutes longer and the cost of surgery was approximately 5000 USD higher in RG than in LG. Thus, the authors concluded that RG has no advantage that counterbalances the time and cost disadvantages.

A single‐center randomized controlled trial recently reported in China has not shown the usefulness of RG over LG in short‐term results.^54^ The slight differences in the content of surgery between Japan and other countries are undeniable, but it is significant to show the reduction of postoperative complication rates as proof of the usefulness of RG. The JCOG is currently conducting a multicenter prospective randomized phase III trial to validate the superiority of RG in terms of reducing the morbidity in JCOG1907 (UMIN000039825). The primary endpoint is the incidence of intra‐abdominal infectious complications, and the sample size is 1040 cases. This trial will reveal the real benefit of RG.

## 9 FUTURE PROSPECTS

In the future, endoscopic surgery is expected to be used for more complicated surgical procedures and to improve prognosis by minimizing surgical invasiveness, even in highly advanced stages of gastric cancer. Furthermore, the introduction of artificial intelligence is expected to help in the development of new surgical procedures. It can be said with some certainty that endoscopic surgery will play a leading role in gastric cancer therapy in the near future. Evidence based on clinical studies must be urgently established to facilitate such advances in endoscopic surgery.

In addition, the efficacy of perioperative chemotherapy has remarkably progressed these days.^55^, ^56^ Immuno‐checkpoint inhibitors were also introduced in the perioperative treatment for gastric cancer. With the progress of systemic chemotherapy, conversion therapy is becoming more and more common.^57^ It has become sometimes possible to curatively resect a tumor that was thought to be unresectable before treatment. The indication and procedure of surgical treatment for advanced gastric cancer may drastically change in the future.

## DISCLOSURES

Conflict of Interest: M.T. reports personal fees from Taiho Pharma, Chugai Pharmaceutical, Ono Pharmaceutical, Bristol‐Myers Squibb, Yakult Honsha, Takeda Pharmaceutical, Eli Lilly Japan, Pfizer Japan, Daiichi‐Sankyo, Johnson and Johnson, Medtronic, Olympus, and Nippon Kayaku, in addition to grants and personal fees from Intuitive Surgical outside the submitted work.

## References

[1] Billroth T . Offenes Schreiben an Herrn Dr. L. Wittelshöfer. Wiener Medizinische Wochenschrift. Band. 1881;31:S161–5.

[2] Billroth T . Über 124 vom November 1878 bis Juni 1890 in meiner Klinik und Privatpraxis ausgeführte Resectionen am Magen‐Darmcanal, Gastro‐Enterostomien und Narbenlosungen wegen chronischer Krankheitsprocesse. Wiener klin Wochenschrift. 1891;4:S625–8.

[3] von Mikulicz‐Radecki J . Bericht über 103 Operationen am Magen. Arch Klin Chir. 1896;51:9–39.

[4] von Mikulicz‐Radecki J . Beitriige zur Technik der Operation des Magencarcino‐ mans. (Contributions to the technique of operating for carcinoma of the stomach.). Arch Klin Chir. 1989;57:524.

[5] Mikulicz‐Radecki V . Surgery of the pancreas. Ann Surg. 1903;38:1–29. 10.1097/00000658-190307000-00001 PMC1431114 17861321

[6] Groves E . On the radical operation for cancer of the pylorus. Br Med J. 1910;12:366–70. 10.1136/bmj.1.2563.366 PMC2330344 20764919

[7] Brunschwig A . Pancreato‐total gastrectomy and splenectomy for advanced carcinoma of the stomach. Cancer. 1948;1:427–30. 18887909 10.1002/1097-0142(194809)1:3<427::aid-cncr2820010308>3.0.co;2-g

[8] Lahey FH , Marshall SF . Should total gastrectomy be employed in eraly carcinoma of the stomach? Experience with 139 total gastrectomies. Ann.Surg. 1950;132:540–60. 15433220 10.1097/00000658-195009000-00019 PMC1616758

[9] Appleby LH . The coeliac axis in the expansion of the operation for gastric carcinoma. Cancer. 1953;6:704–7. 13059764 10.1002/1097-0142(195307)6:4<704::aid-cncr2820060410>3.0.co;2-p

[10] Lawrence W Jr , McNeer G . An analysis of the role of radical surgery for gastric cancer. Surg Gynecol Obstet. 1960;111:691–6. 13759707

[11] Fisher B . Laboratory and clinical research in breast cancer–a personal adventure: the David A. Karnofsky Memorial Lecture. Cancer Res. 1980;40:3863–74. 7008932

[12] Fisher B , Redmond C , Fisher ER , Bauer M , Wolmark N , Wickerham DL , et al. Ten‐year results of a randomized clinical trial comparing radical mastectomy and total mastectomy with or without radiation. N Engl J Med. 1985;312:674–81. 3883168 10.1056/NEJM198503143121102

[13] Kajitani T . The general rules for the gastric cancer study in surgery and pathology. Part I. Clinical classification. Jpn J Surg. 1981;11:127–39. 7300058 10.1007/BF02468883

[14] Isozaki H , Okajima K , Fujii K , Nomura E , Izumi N , Mabuchi H , et al. Effectiveness of paraaortic lymph node dissection for advanced gastric cancer. Hepatogastroenterology. 1999;46:549–54. 10228860

[15] Cuschieri A , Weeden S , Fielding J , Bancewicz J , Craven J , Joypaul V , et al. Patient survival after D1 and D2 resections for gastric cancer: long‐term results of the MRC randomized surgical trial. Br J Cancer. 1999;79(9–10):1522–30. 10188901 10.1038/sj.bjc.6690243 PMC2362742

[16] Bonenkamp JJ , Hermans J , Sasako M , Welvaart K , Songun I , Meyer S , et al. Extended lymph‐node dissection for gastric cancer. N Engl J Med. 1999;40(12):908–14. 10.1056/NEJM199903253401202 10089184

[17] Sasako M , Sano T , Yamamoto S , Kurokawa Y , Nashimoto A , Kurita A , et al. D2 lymphadenectomy alone or with para‐aortic nodal dissection for gastric cancer. N Engl J Med. 2008;359(5):453–62. 18669424 10.1056/NEJMoa0707035

[18] Harter P , Sehouli J , Lorusso D , Reuss A , Vergote I , Marth C , et al. A randomized trial of lymphadenectomy in patients with advanced ovarian neoplasms. N Engl J Med. 2019;380(9):822–32. 30811909 10.1056/NEJMoa1808424

[19] Songun I , Putter H , Kranenbarg EM , Sasako M , van de Velde CJ . Surgical treatment of gastric cancer: 15‐year follow‐up results of the randomised nationwide Dutch D1D2 trial. Lancet Oncol. 2010;11(5):439–49. 20409751 10.1016/S1470-2045(10)70070-X

[20] Sano T , Sasako M , Mizusawa J , Yamamoto S , Katai H , Yoshikawa T , et al. Randomized controlled trial to evaluate splenectomy in total gastrectomy for proximal gastric carcinoma. Ann Surg. 2017;265(2):277–83. 27280511 10.1097/SLA.0000000000001814

[21] Watanabe M , Kinoshita T , Enomoto N , Shibasaki H , Nishida T . Clinical significance of splenic Hilar dissection with splenectomy in advanced proximal gastric cancer: an analysis at a single institution in Japan. World J Surg. 2016;40(5):1165–71. 26630939 10.1007/s00268-015-3362-4

[22] Maezawa Y , Aoyama T , Yamada T , Kano K , Hayashi T , Sato T , et al. Priority of lymph node dissection for proximal gastric cancer invading the greater curvature. Gastric Cancer. 2018;21(3):569–72. 29119277 10.1007/s10120-017-0775-9

[23] Huang CM , Huang ZN , Zheng CH , Li P , Xie JW , Wang JB , et al. Huang's three‐step maneuver shortens the learning curve of laparoscopic spleen‐preserving splenic hilar lymphadenectomy. Surg Oncol. 2017;26(4):389–94. 29113657 10.1016/j.suronc.2017.07.010

[24] Sasako M , Sano T , Yamamoto S , Sairenji M , Arai K , Kinoshita T , et al. Left thoracoabdominal approach versus abdominal‐transhiatal approach for gastric cancer of the cardia or subcardia: a randomised controlled trial. Lancet Oncol. 2006;7(8):644–51. 16887481 10.1016/S1470-2045(06)70766-5

[25] Kurokawa Y , Doki Y , Mizusawa J , Terashima M , Katai H , Yoshikawa T , et al. Bursectomy versus omentectomy alone for resectable gastric cancer (JCOG1001): a phase 3, open‐label, randomised controlled trial. Lancet Gastroenterol Hepatol. 2018;3:460–8. 29709558 10.1016/S2468-1253(18)30090-6

[26] Tokunaga M , Tanizawa Y , Bando E , Kawamura T , Terashima M . Poor survival rate in patients with postoperative intra‐abdominal infectious complications following curative gastrectomy for gastric cancer. Ann Surg Oncol. 2013;20(5):1575–83. 23076557 10.1245/s10434-012-2720-9

[27] Shimada H , Fukagawa T , Haga Y , Oba K . Does postoperative morbidity worsen the oncological outcome after radical surgery for gastrointestinal cancers? A systematic review of the literature. Ann Gastroenterol Surg. 2017;1(1):11–23. 29863169 10.1002/ags3.12002 PMC5881350

[28] Kitano S , Iso Y , Moriyama M , Sugimachi K . Laparoscopy‐assisted Billroth I gastrectomy. Surg Laparosc Endosc. 1994;4(2):146–8. 8180768

[29] Katai H , Sasako M , Fukuda H , Nakamura K , Hiki N , Saka M , et al. Safety and feasibility of laparoscopy‐assisted distal gastrectomy with suprapancreatic nodal dissection for clinical stage I gastric cancer: a multicenter phase II trial (JCOG 0703). Gastric Cancer. 2010;13(4):238–44. 21128059 10.1007/s10120-010-0565-0

[30] Katai H , Mizusawa J , Katayama H , Morita S , Yamada T , Bando E , et al. Survival outcomes after laparoscopy‐assisted distal gastrectomy versus open distal gastrectomy with nodal dissection for clinical stage IA or IB gastric cancer (JCOG0912): a multicentre, noninferiority, phase 3 randomised controlled trial. Lancet Gastroenterol Hepatol. 2020;5(2):142–51. 31757656 10.1016/S2468-1253(19)30332-2

[31] Kim HH , Han SU , Kim MC , Kim W , Lee HJ , Ryu SW , et al. Effect of laparoscopic distal gastrectomy vs open distal gastrectomy on long‐term survival among patients with stage I gastric cancer: the KLASS‐01 randomized clinical trial. JAMA Oncol. 2019;5(4):506–13. 30730546 10.1001/jamaoncol.2018.6727 PMC6459124

[32] Katai H , Mizusawa J , Katayama H , Kunisaki C , Sakuramoto S , Inaki N , et al. Single‐arm confirmatory trial of laparoscopy‐assisted total or proximal gastrectomy with nodal dissection for clinical stage I gastric cancer: Japan Clinical Oncology Group study JCOG1401. Gastric Cancer. 2019;22(5):999–1008. 30788750 10.1007/s10120-019-00929-9

[33] Hyung WJ , Yang HK , Han SU , Lee YJ , Park JM , Kim JJ , et al. A feasibility study of laparoscopic total gastrectomy for clinical stage I gastric cancer: a prospective multi‐center phase II clinical trial, KLASS 03. Gastric Cancer. 2019;22(1):214–22. 30128720 10.1007/s10120-018-0864-4

[34] Yu J , Huang CM , Sun YH , Xiangqian S , Cao H , Jiankun H , et al. Effect of laparoscopic vs open distal gastrectomy on 3‐year disease‐free survival in patients with locally advanced gastric cancer. The CLASS‐01 randomized clinical trial. JAMA. 2019;321:1983–92. 31135850 10.1001/jama.2019.5359 PMC6547120

[35] Hyung WJ , Yang HK , Park YK , Lee HJ , An JY , Kim W , et al. Long‐term outcomes of laparoscopic distal gastrectomy for locally advanced gastric cancer: The KLASS‐02‐RCT randomized clinical trial. J Clin Oncol. 2020;38:3304–13. 32816629 10.1200/JCO.20.01210

[36] Etoh T , Shiroshita H , Shiraishi N , Kitano S , Inomata M . Ongoing clinical studies of minimally invasive surgery for gastric cancer in Japan. Transl Gastroenterol Hepatol. 2016;1:31–7. 28138598 10.21037/tgh.2016.03.15 PMC5244702

[37] Haverkamp L , Brenkman HJ , Seesing MF , Gisbertz SS , van Berge MIH , Luyer MDP , et al. Laparoscopic versus open gastrectomy for gastric cancer, a multicenter prospectively randomized controlled trial (LOGICA‐trial). BMC Cancer. 2015;15:556–63. 26219670 10.1186/s12885-015-1551-z PMC4518687

[38] van der Wielen N , Straatman J , Daams F , Rosati R , Parise P , Weitz J , et al. Open versus minimally invasive total gastrectomy after neoadjuvant chemotherapy: results of a European randomized trial. Gastric Cancer. 2020;24(1):258–71. 10.1007/s10120-020-01109-w. Online ahead of print. 32737637 PMC7790799

[39] Yoshida K , Honda M , Kumamaru H , Kodera Y , Kakeji Y , Hiki N , et al. Surgical outcomes of laparoscopic distal gastrectomy compared to open distal gastrectomy: a retrospective cohort study based on a nationwide registry database in Japan. Ann Gastroenterol Surg. 2017;2(1):55–64. 29863131 10.1002/ags3.12054 PMC5881294

[40] Hiki N , Honda M , Etoh T , Yoshida K , Kodera Y , Kakeji Y , et al. Higher incidence of pancreatic fistula in laparoscopic gastrectomy. Real‐world evidence from a nationwide prospective cohort study. Gastric Cancer. 2018;21(1):162–70. 28887712 10.1007/s10120-017-0764-z

[41] Kodera Y , Yoshida K , Kumamaru H , Kakeji Y , Hiki N , Etoh T , et al. Introducing laparoscopic total gastrectomy for gastric cancer in general practice: a retrospective cohort study based on a nationwide registry database in Japan. Gastric Cancer. 2019;22(1):202–13. 29427039 10.1007/s10120-018-0795-0

[42] Etoh T , Honda M , Kumamaru H , Miyata H , Yoshida K , Kodera Y , et al. Morbidity and mortality from a propensity score‐matched, prospective cohort study of laparoscopic versus open total gastrectomy for gastric cancer: data from a nationwide web‐based database. Surg Endosc. 2018;32(6):2766–73. 29218676 10.1007/s00464-017-5976-0

[43] Hashizume M , Shimada M , Tomikawa M , Ikeda Y , Takahashi I , Abe R , et al. Early experiences of endoscopic procedures in general surgery assisted by a computer‐enhanced surgical system. Surg Endosc. 2002;16(8):1187–91. 11984681 10.1007/s004640080154

[44] Kakeji Y , Konishi K , Ieiri S , Yasunaga T , Nakamoto M , Tanoue K , et al. Robotic laparoscopic distal gastrectomy: a comparison of the da Vinci and Zeus systems. Int J Med Robot. 2006;2(4):299–304. 17520647 10.1002/rcs.104

[45] Chen K , Pan Y , Zhang B , Maher H , Wang XF , Cai XJ . Robotic versus laparoscopic Gastrectomy for gastric cancer: a systematic review and updated meta‐analysis. BMC Surg. 2017;17(1):93–107. 28836986 10.1186/s12893-017-0290-2 PMC5571509

[46] Caruso S , Patriti A , Roviello F , De Franco L , Franceschini F , Ceccarelli G , et al. Robot‐assisted laparoscopic vs open gastrectomy for gastric cancer: systematic review and meta‐analysis. World J Clin Oncol. 2017;8(3):273–84. 28638798 10.5306/wjco.v8.i3.273 PMC5465018

[47] Bobo Z , Xin W , Jiang L , Quan W , Liang B , Xiangbing D , Ziqiang W , et al. Robotic gastrectomy versus laparoscopic gastrectomy for gastric cancer: meta‐analysis and trial sequential analysis of prospective observational studies. Surg Endosc. 2019;33(4):1033–48. 30719561 10.1007/s00464-018-06648-z

[48] Tokunaga M , Sugisawa N , Kondo J , Tanizawa Y , Bando E , Kawamura T , Terashima M , et al. Early phase II study of robot‐assisted distal gastrectomy with nodal dissection for clinical stage IA gastric cancer. Gastric Cancer. 2014;17(3):542–7. 24005955 10.1007/s10120-013-0293-3

[49] Tokunaga M , Makuuchi R , Miki Y , Tanizawa Y , Bando E , Kawamura T , Terashima M , et al. Late phase II study of robot‐assisted gastrectomy with nodal dissection for clinical stage I gastric cancer. Surg Endosc. 2016;30(8):3362–7. 26511119 10.1007/s00464-015-4613-z

[50] Shibasaki S , Suda K , Nakauchi M , Nakamura K , Kikuchi K , Inaba K , Uyama I , et al. Non‐robotic minimally invasive gastrectomy as an independent risk factor for postoperative intra‐abdominal infectious complications: a single‐center, retrospective and propensity score‐matched analysis. World J Gastroenterol. 2020;26(11):1172–84. 32231421 10.3748/wjg.v26.i11.1172 PMC7093317

[51] Guerrini GP , Esposito G , Magistri P , Serra V , Guidetti C , Olivieri T , et al. Robotic versus laparoscopic gastrectomy for gastric cancer: the largest meta‐analysis. Int J Surg. 2020;82:210–28. 32800976 10.1016/j.ijsu.2020.07.053

[52] Uyama I , Suda K , Nakauchi M , Kinoshita T , Noshiro H , Takiguchi S , et al. Clinical advantages of robotic gastrectomy for clinical stage I/II gastric cancer: a multi‐institutional prospective single‐arm study. Gastric Cancer. 2019;22(2):377–85. 30506394 10.1007/s10120-018-00906-8

[53] Kim HI , Han SU , Yang HK , Kim YW , Lee HJ , Ryu KW , et al. Multicenter prospective comparative study of robotic versus laparoscopic gastrectomy for gastric adenocarcinoma. Ann Surg. 2016;263(1):103–9. 26020107 10.1097/SLA.0000000000001249

[54] Lu J , Zheng CH , Xu BB , Xie JW , Wang JB , Lin JX , et al. Assessment of robotic versus laparoscopic distal gastrectomy for gastric cancer. Ann Surg. 2020. 10.1097/SLA.0000000000004466. Online ahead of print. 32889876

[55] Terashima M , Yoshikawa T , Boku N , Ito S , Tsuburaya A , Iwasaki Y , et al. Current status of perioperative chemotherapy for locally advanced gastric cancer and JCOG perspectives. Jpn J Clin Oncol. 2020;50:528–34. 32134452 10.1093/jjco/hyaa005

[56] Wagner AD , Lordick F , Terashima M , Terada M , Yoshikawa T , Boku N , et al. Multidisciplinary management of stage II‐III gastric and gastro‐oesophageal junction cancer. Eur J Cancer. 2020;124:67–76. 10.1016/j.ejca.2019.09.006 31759294

[57] Terashima M . Conversion therapy for gastric cancer: who can make conversion as successful as Goromaru? Gastric Cancer. 2016;19:685–6. 27055560 10.1007/s10120-016-0609-1

